# Is *Sellaphora* the New *Navicula*? *Cymbosellaphora* (Cymbellales), a New Genus Based on Taxa Previously Assigned to *Sellaphora*

**DOI:** 10.3390/plants12223890

**Published:** 2023-11-17

**Authors:** Maxim S. Kulikovskiy, Anton M. Glushchenko, Sergei I. Genkal, Irina V. Kuznetsova, Yevhen I. Maltsev, John Patrick Kociolek

**Affiliations:** 1 K.A. Timiryazev Institute of Plant Physiology, Russian Academy of Sciences (IPP RAS), 35 Botanicheskaya St., 127276 Moscow, Russiapantao@yandex.ru (I.V.K.); ye.maltsev@gmail.com (Y.I.M.); 2 Papanin Institute for Biology of Inland Waters, Russian Academy of Sciences (IBIW RAS), Nekouz District, Yaroslavl Region, 152742 Borok, Russia; genkal47@mail.ru; 3 Museum of Natural History, Department of Ecology and Evolutionary Biology, University of Colorado, Henderson Building, 15th and Broadway, Boulder, CO 80309, USA

**Keywords:** diatoms, *Navicula*, *Sellaphora*, *Cymbella*, *Cymbosellaphora*, new genus, morphology

## Abstract

A new diatom genus *Cymbosellaphora* Kulikovskiy, Glushchenko, Genkal and Kociolek gen. nov., was described with species *Cymbosellaphora vietnamensis* Glushchenko, Kulikovskiy and Kociolek sp. nov. *C. vietnamensis* sp. nov. was described from Vietnam and characterized by the presence of morphological features such as valves with naviculoid symmetry, slight dorsiventrality, the presence of tectula as pore occlusions, uniseriate striae, and a very broad mantle. Four species were transferred to the new genus. These are *C. absoluta* comb. nov., *C. circumborealis* comb. nov., *C. geisslerae* comb. nov., and *C. laterostrata* comb. nov. Previously, these species were members of genera *Navicula* Bory, *Sellaphora* Mereschkowsky, and *Naviculadicta* Lange-Bertalot. The taxonomic history of these species and genera are discussed. The tectulum is known only from the cymbelloid diatoms, and our new genus is placed within the Cymbellaceae. The presence of a tectulum demonstrates that these species cannot be placed in *Sellaphora*, as indicated in the literature. The recent proposal to transfer a large number of species with different morphologies to the genus *Sellaphora* is also discussed. Additionally, we compare pore occlusions with tectula between different genera of the Cymbellaceae with naviculoid symmetry.

## 1. Introduction

Wetzel et al. [1] recently proposed more than 60 new taxonomical combinations in the genus *Sellaphora* Mereschkowsky. They discussed the morphology and taxonomy of many small-celled naviculoid diatoms that play important roles in ecological analyses; many of these taxa are widespread. New combinations were proposed, transferring many species previously known to be members of *Navicula* Bory, *Naviculadicta* Lange-Bertalot, and *Eolimna* Lange-Bertalot and Schiller to *Sellaphora*. Wetzel et al. [1] comprehensively investigated the type material of some species with light and scanning electron microscopy. However, some new combinations were proposed without investigating morphology with the benefit of SEM, and without referring to published observations where SEM investigations were conducted. Examples include *Sellaphora absoluta* (Hustedt) C.E. Wetzel, Ector, Van de Vijver, Compere and D.G. Mann; and *Sellaphora circumborealis* (Lange-Bertalot) C.E. Wetzel, Ector, Van de Vijver, Compere and D.G. Mann. As a result, genera such as *Naviculadicta* and *Eolimna* were left without taxa, and the genus *Sellaphora* grew dramatically, going on to be one of the largest freshwater naviculoid genera.

The genus *Sellaphora* includes species that differ in size and also such morphological features as presence or absence of conopeum, longitudinal grooves, apical pits, and uniseriate or biseriate striae, among others [2,3]. *Sellaphora* was established by Mereschkowsky [4] based on the presence of an H-shaped plastid containing an invaginated pyrenoid. D.G. Mann and his colleagues have provided comprehensive investigations of the silica valve structure, as well as the chloroplast structure, sexual reproduction, and mating system [2,5,6,7,8,9,10]. According to above-mentioned investigations and morphological description given by Round et al. [11] and Wetzel et al. [1], *Sellaphora*, as a genus, is characterized by naviculoid and solitary cells with a number of morphological features, including the following:

(1) Striae that are uniseriate or biseriate, radiate, or parallel;
(2) A nonporous conopeum that can be present adjacent to the axial area;
(3) A flat valve face that curves fairly gently into shallow or moderately deep mantles;
(4) Striae that contain small round poroids, which are occluded near their internal apertures by hymenes;
(5) A central and straight raphe system, with distal raphe ends deflected or hooked;
(6) A central external raphe endings that are expanded and slightly deflected towards the primary side;
(7) A central internal raphe endings turned or deflected towards the primary side;
(8) The presence of helictoglossae.

This concept of *Sellaphora* describes the genus as a typical naviculoid taxon with a centrally positioned filiform raphe, having uniseriate or biseriate striae with pores that are covered internally. This description differentiates *Sellaphora* from the genus *Eolimna*, which has large areolae (relative to cell size) occluded by hymenes positioned medially in the areolae. Comparison with the genus *Naviculadicta* is not productive because this genus was created during a period of taxonomical instability related to naviculoid diatom genera, and it was suggested that it was only a matter of time until species would be transferred from this artificial group into a natural group [12]. Molecular studies of species from the genus [13,14,15,16,17] have suggested that *Sellaphora* possibly represents a monophyletic group that also includes *Eolimna* Lange-Bertalot and Schiller in Schiller et Lange-Bertalot [18] and *Diprora* Main [19]. However, the number of species used in these phylogenetic reconstructions is not large, with only 17 having been identified to the species level, 4 taxa having been identified presumptively, and 18 not having been identified [20].

Morphological investigations of diatoms continue to be important to understanding taxonomic identifications and for assessing phylogenetic relationships. Taxonomical investigations based on morphology are important because diatoms play important roles in the ecological monitoring of aquatic ecosystems, stratigraphy, and many other applied disciplines [1]. Different morphological features play different roles in diatom taxonomy [21] since they can diagnose groups at different levels in the hierarchy of classification according to formal analyses of morphological data.

Pore occlusions have been used to delineate taxonomic groups at the Family and Order levels [12]. Mann [22], who discussed a phenetic approach to diatom systematics, discussed the important role of pore occlusion in understanding of diatom phylogeny. Similar pore occlusions should characterize “natural” monophyletic groups, and these groups should share the same pore occlusion types—an idea that was later supported by Cox [23]. Widely, three velum types (cribrum, rota, and vola) have been used for describing pore occlusions in pennate diatoms [11,24,25]. Later, hymenate occlusions were described by D.G. Mann [22]. He described hymenes as tiny and slim perforations that are dappled by very small (in many cases) circular holes with a diameter less than 5 nm or 5–10 nm (see Round et al.) [11]. Hymenes are mainly known in members of the Order Naviculales Bessey 1907 sensu emend. Round, Crawford and D.G. Mann 1900 [12].

Cox [26] discussed and presented a reassessment of the structure and terminology of pore occlusions in the members of the Cymbellales with the description of two new types of pore occlusions, namely, tectula and foricula. Cox [23] described foricula as a “thinner flange-like outgrowth with a wide point of attachment, which partially occludes an areola, usually on the outer surface” and tectulum as “a round or squarish flap-like covering, attached to the edges of an areola by several, regularly arranged small struts”. According to Cox [23], foricula are known from members of *Cymbella* Agardh sensu stricto; *Gomphonema* Ehrenberg; *Gomphoneis* P.T. Cleve; and *Reimeria* Kociolek and Stoermer. Tectula, according to Cox [23], are known in the genus *Placoneis* Mereschkowsky. Based on these observations, we can distinguish naviculoid diatom genera from cymbelloid on the basis of pore occlusion.

The aim of this publication is to provide additional morphological evidence of some naviculoid species, previously belonging to the genera *Sellaphora*, *Navicula*, and *Naviculadicta*, and, based on the results of this study, to describe the genus *Cymbosellaphora* gen. nov.

## 2. Results and Discussion

***Cymbosellaphora* Kulikovskiy, Glushchenko, Genkal and Kociolek gen. nov.**

Type species (designated here): *Cymbosellaphora vietnamensis* Glushchenko, Kulikovskiy and Kociolek sp. nov.

**Description**

**LM.** Valves nearly linear–elliptic to almost rhombic–lanceolate. Valves naviculoid or slightly dorsiventral. Ends broadly protracted and broadly rounded, obtusely rounded, sometimes rostrate or sub-capitate. Striae uniseriate, parallel or slightly radiate, radiate in the center, convergent near the ends, puncta usually resolvable. Axial area narrow, straight. Central area variable, small-to-medium size, transversely widened and irregularly delimited. Raphe straight and filiform. Central raphe ends straight and puncta-like, curved to opposite from distal raphe end side. Distal raphe ends hooked, extending onto the mantle.

**SEM, external view.** Valve face flat without visible sternum. Raphe lying in the middle of the valve. Central raphe ends straight, point-like and slightly curved to the side opposite from the distal raphe ends. Central nodule broad. Distal raphe ends hook-like and curved to mantle. Striae uniseriate with distantly spaced circle or linear, zig-zag linear areolae. Striae at both ends are regularly spaced or slightly approximating. Pore fields not detected. Mantle very wide with striae and areolae continuing from valve face. Girdle bands with two rows of perforations.

**SEM, internal view.** Axial area straight and with narrow sternum evident or slightly evident. Raphe with curved central raphe ends, distal raphe ends straight and finished by evident helictoglossae. Central nodule broad. Interstriae not elevated, wide and broader than striae. Striae uniseriate and includes areolae. Areolae covered with a thin imperforate flat silica layer. This silica layer covers the areolae at one level with the inner surface of the valve. When this layer is destroyed, the internal structure of the areola becomes visible. Every one hole has two levels. At one level, the opening is broader and circular or rectangular, and on this level, a silica layer that covers areola internally is present. The second level has a smaller hole. These smaller holes are circular or linear and extend through the valve. Every one circle or circular areola is divided by rectangular strut. During the cleaning of the valves, these rectangular struts are destroyed and are represented by two outgrowths situated parallel to the valve level and opposite one another. These struts or tectula are present between areolar openings within each stria. Voigt discontinuity is present on the secondary side of the valves.

**Etymology:** This genus name is a combination of the genus names *Sellaphora* and *Cymbella*, indicating that they share some similar morphological features of both genera.

**Figure 1:**
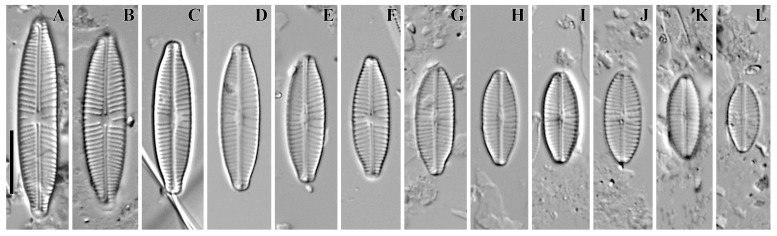
(**A**–**L**) *Cymbosellaphora vietnamensis* Glushchenko, Kulikovskiy and Kociolek sp. nov. Light microscopy, differential interference contrast, size diminution series. Vietnam. Slide no. 02151. (**A**) Holotype. Scale bar = 10 μm.

**Figure 2:**
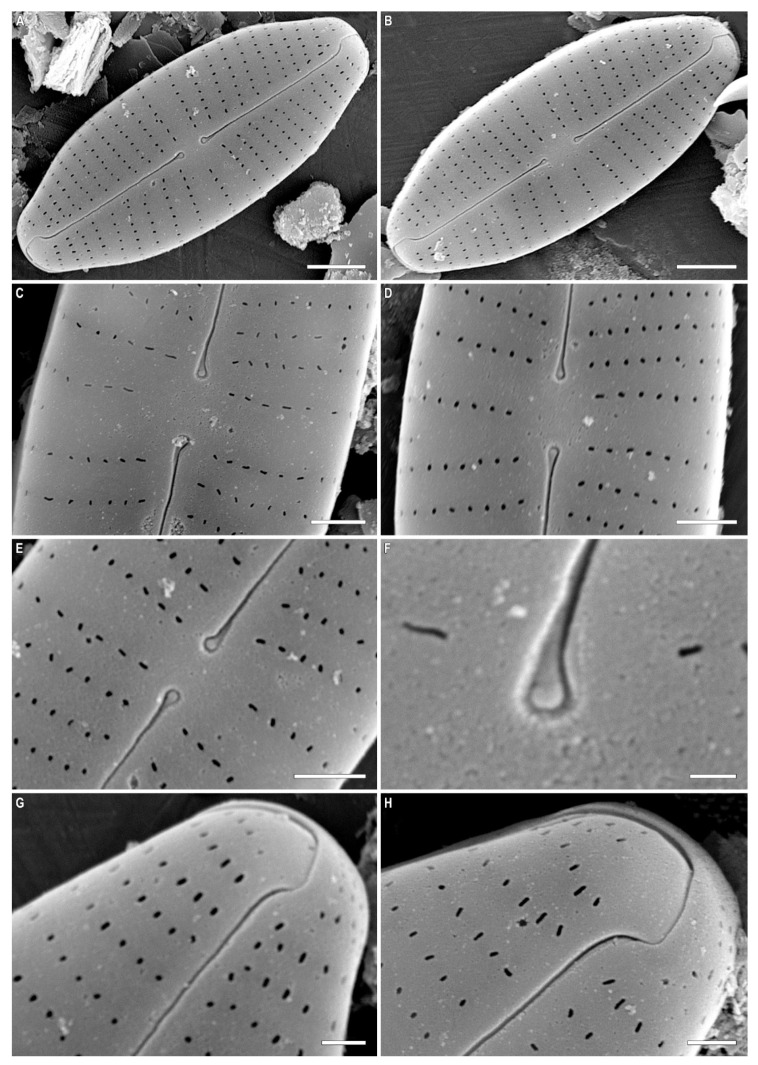
(**A**–**H**) *Cymbosellaphora vietnamensis* Glushchenko, Kulikovskiy and Kociolek sp. nov. Scanning electron microscopy, external views. (**A**,**B**) The whole valve. (**C**–**E**) Central area. (**F**) Central raphe end. (**G**,**H**) Valve ends. Scale bar (**A**,**B**) = 2 μm; (**C**–**E**) = 1 μm; (G,H) = 0.5 μm (**F**) = 0.2 μm.

**Figure 3:**
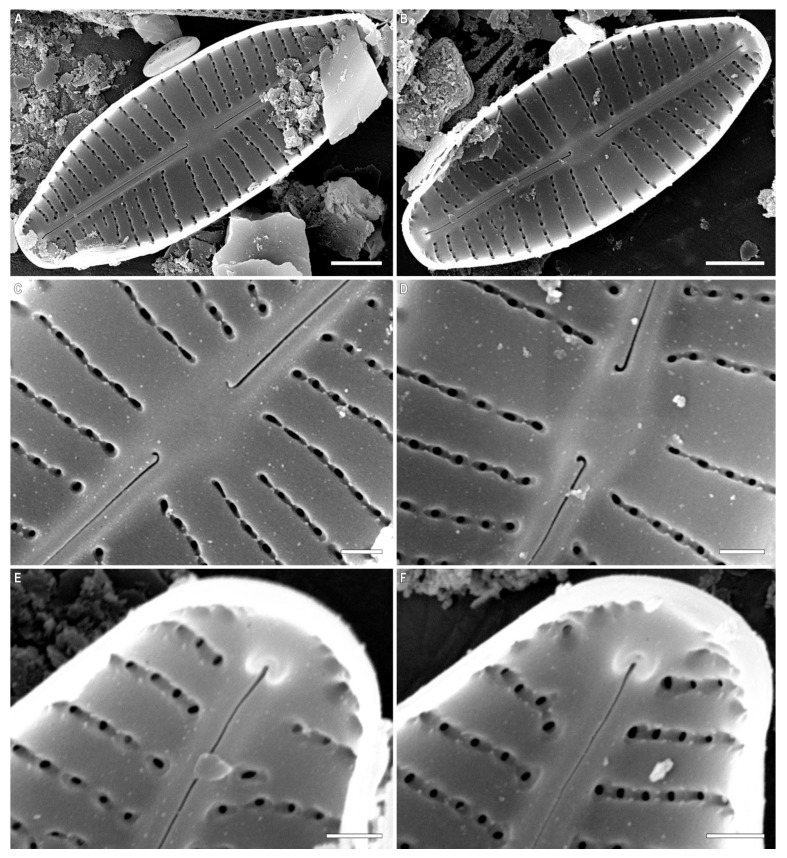
(**A**–**F**) *Cymbosellaphora vietnamensis* Glushchenko, Kulikovskiy and Kociolek sp. nov. Scanning electron microscopy, internal views. (**A**,**B**) The whole valve. (**C**,**D**) Central area. (**E**,**F**) Valve ends. Tectula, as small struts, are present between areolar openings within each stria. Voigt discontinuity is clearly visible. Scale bar (**A**,**B**) = 2 μm; (**C**–**F**) = 0.5 μm.

***Cymbosellaphora vietnamensis* Glushchenko, Kulikovskiy and Kociolek sp. nov. (Figure 1, Figure 2 and Figure 3)**

**Holotype.** Deposited in the herbarium of MHA, Main Botanical Garden Russian Academy of Science, Moscow, Russia, the holotype here designated, slide no. 12151 (Figure 1A).

**Isotype.** Collection of Maxim Kulikovskiy at the Herbarium of the Institute of Plant Physiology Russian Academy of Science, Moscow, Russia, slide no. 12151a.

**Etymology:** The specific epithet “*vietnamensis*” refers to the name of the country where this species was discovered.

**Description. LM (Figure 1A–L).** Valves almost linear to linear–elliptic. Valves naviculoid, slightly dorsiventral in longer specimens. Length 10.7–31.4 µm; breadth 5.0–6.7 µm. Ends broadly protracted. Axial area narrow, straight. Central area variable, small-to-medium size, transversely widened and irregularly delimited. Raphe straight and filiform. Central raphe ends straight and puncta-like, curved to opposite from distal raphe end side. Distal raphe ends hooked, going to the mantle. Striae uniseriate, slightly radiate, convergent near the ends, 14–16 in 10 µm. Areolae noticeable in some specimens.

**SEM, external view (Figure 2A–F).** Valve face flat without visible sternum. Raphe positioned in the middle of the valve. Central raphe ends straight, point-like, and slightly curved to the side opposite the distal raphe ends. Central nodule broad. Distal raphe ends hook-like and curved onto the mantle. Striae uniseriate with distantly spaced circular or linear areolae. Striae at both ends are regularly spaced or slightly approximating. Pore fields not detected. Mantle very wide with striae and areolae continuing from valve face. Areolae 35–40 in 10 µm.

**SEM, internal view (Figure 3A–F).** Axial area straight and present by evident or slightly evident narrow sternum. Raphe with curved central raphe ends, distal raphe ends straight, and they terminate as evident helictoglossae. Central nodule broad. Interstriae not elevated, wide and broader than striae. Striae uniseriate. Areolae covered by a thin imperforate flat silica layer. This silica layer covers the areolae at one level with the inner surface of the valve. When this layer is destroyed, the internal structure of the areola becomes visible. Every opening has two levels. At one level, the opening is broader and circular or rectangular. On this level, a silica layer is present that covers areolae internally. Voigt discontinuity is present on the secondary side of the valves.

The openings at the second level have a smaller aperture, are circular or linear in shape, and extend through the valves. Every areola is divided by a rectangular strut. During the digestion of valve, these rectangular struts can be destroyed, and what is revealed are two outgrowths situated parallel to valve level and opposite one another.

**New combinations:**

**Figure 4:**
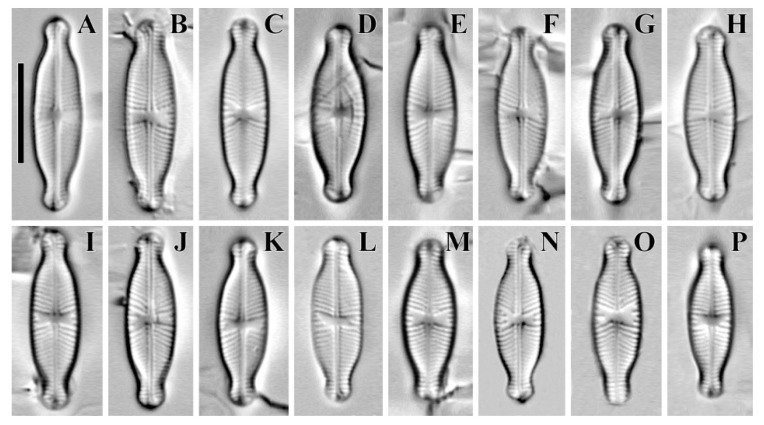
(**A**–**P**) *Cymbosellaphora absoluta* Kulikovskiy, Glushchenko, Genkal and Kociolek comb. nov. Light microscopy, differential interference contrast, size diminution series. Mongolia. Slide no. 02431 (**L**,**P**); 02440 (**A**,**J**); 02441 (**C**,**M**); 02442 (**E**); 02445 (**H**); 02446 (**K**); 02447 (**B**,**F**,**N**,**O**); 02451 (**I**); 02457 (**D**,**G**). Scale bar = 10 μm.

**Figure 5:**
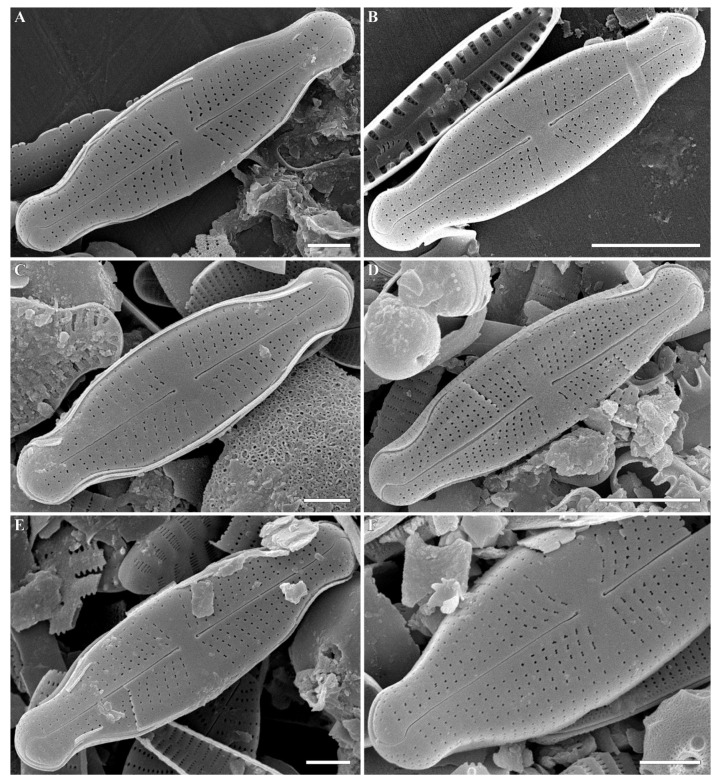
(**A**–**F**) *Cymbosellaphora absoluta* Kulikovskiy, Glushchenko, Genkal and Kociolek comb. nov. Scanning electron microscopy, external views. (**A**–**E**) The whole valve. (**F**) Fragment of valve. Scale bar (**B**,**D**) = 5 μm; (**A**,**C**,**E**,**F**) = 2 μm.

**Figure 6:**
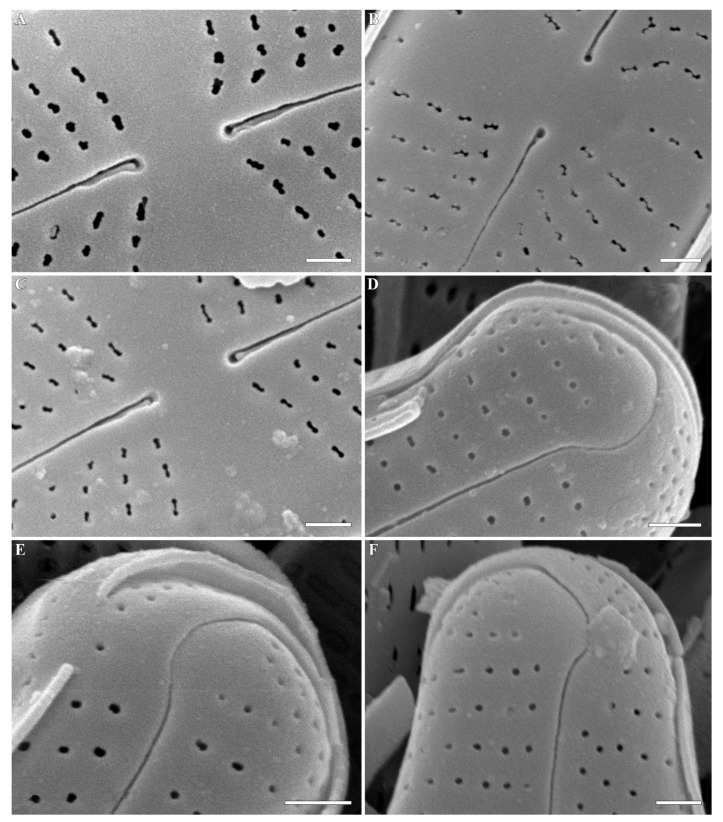
(**A**–**F**) *Cymbosellaphora absoluta* Kulikovskiy, Glushchenko, Genkal and Kociolek comb. nov. Scanning electron microscopy, external views. (**A**–**C**) Central area. (**D**–**F**) Valve ends. Scale bar (**A**–**F**) = 0.5 μm.

**Figure 7:**
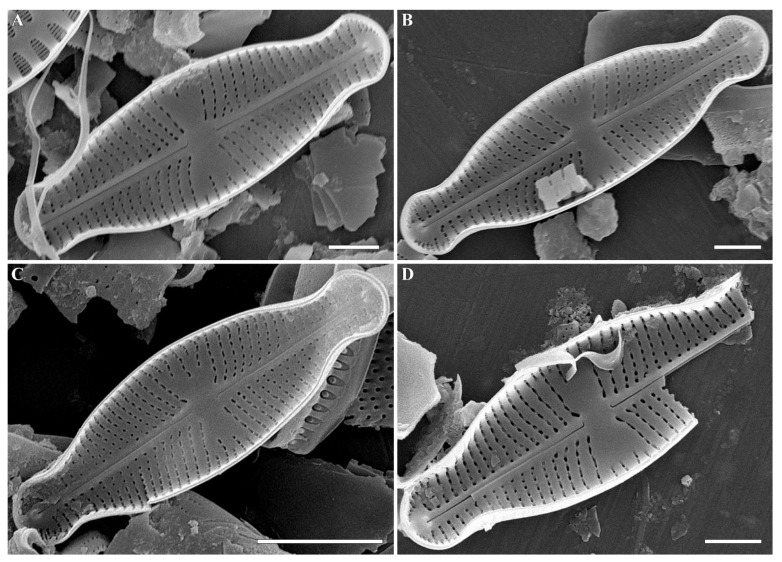
(**A**–**D**) *Cymbosellaphora absoluta* Kulikovskiy, Glushchenko, Genkal and Kociolek comb. nov. Scanning electron microscopy, internal views. (**A**–**C**) The whole valve. Voigt discontinuity is clearly visible. (**D**) Fragment of valve. Scale bar (**C**) = 5 μm; (**A**,**B**,**D**) = 2 μm.

**Figure 8:**
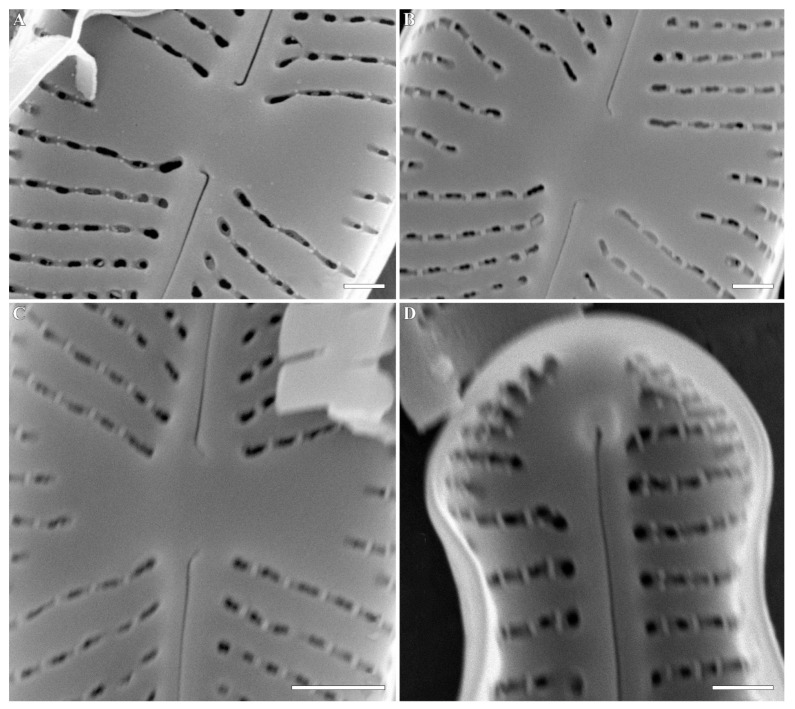
(**A**–**D**) *Cymbosellaphora absoluta* Kulikovskiy, Glushchenko, Genkal and Kociolek comb. nov. Scanning electron microscopy, internal views. (**A**–**C**) Central area. (**D**) Valve end. Tectula, as struts, are present between areolar openings within each stria. Scale bar (**C**) = 1 μm; (**A**,**B**,**D**) = 0.5 μm.

*Cymbosellaphora absoluta* (Hustedt) Kulikovskiy, Glushchenko, Genkal and Kociolek comb. nov. **(Figure 4, Figure 5, Figure 6, Figure 7 and Figure 8)**

**Basionym:** *Navicula absoluta* Hustedt 1950. Archiv für Hydrobiologie. Band 43, P. 435, Pl. 38, Fig. 80–85.

**Synonyms:** *Naviculadicta absoluta* (Hustedt) Lange-Bertalot in Moser and Lange-Bertalot 1994; *Sellaphora absoluta* (Hustedt) Wetzel, Ector, Van de Vijver, Compère and D.G. Mann 2015.

**Comments:** The specimens in our sample were 15.4–19.2 μm long, 4.5–5.5 μm wide, and had 25–26 striae in 10 μm and 25–30 areolae in 10 μm.

**Figure 9:**
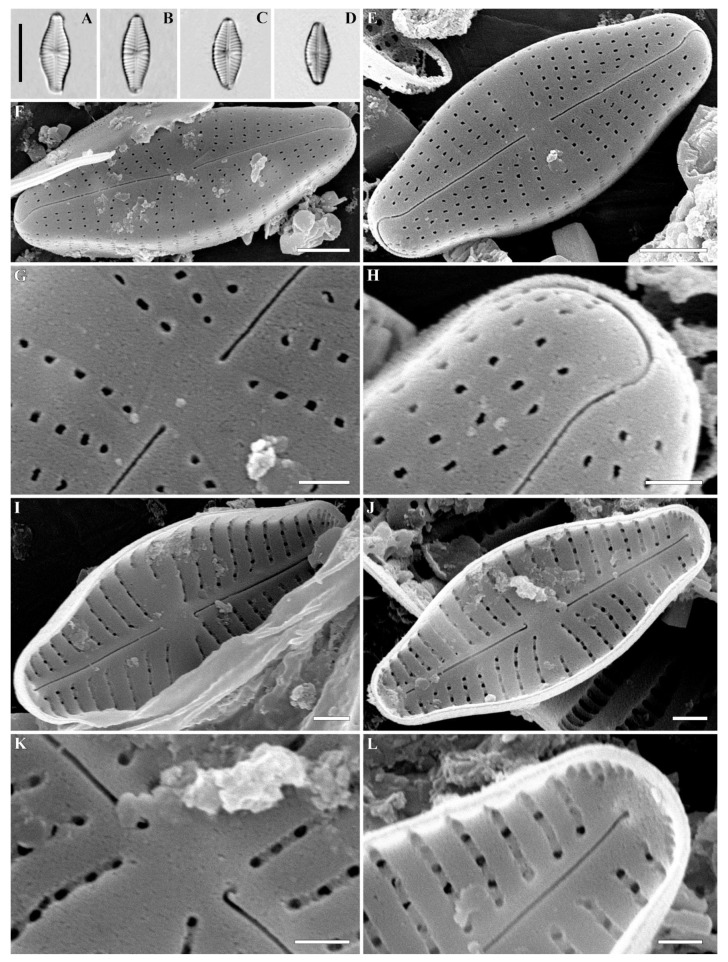
(**A**–**L**) *Cymbosellaphora circumborealis* Kulikovskiy, Glushchenko, Genkal and Kociolek comb. nov. (**A**–**D**) Light microscopy, differential interference contrast, size diminution series. Mongolia. Slide no. 02663 (**B**); 02684 (**A**); 02695 (**C**); 03009 (**D**). (**E**–**H**) SEM, external view. (**I**–**L**) SEM, internal view. (**E**,**F**,**I**,**J**) The whole valve. (**G**,**K**) Central area. (**H**,**L**) Valve ends. Scale bar = (**A**–**D**) = 10 μm; (**E**,**F**) = 2 μm; (**I**,**J**) = 1 μm; (**G**,**H**,**K**,**L**) = 0.5 μm.

*Cymbosellaphora circumborealis* (Lange-Bertalot) Kulikovskiy, Glushchenko, Genkal and Kociolek comb. nov. **(Figure 9)**

**Basionym:**  *Naviculadicta circumborealis* Lange-Bertalot in Lange-Bertalot and Moser 1994. Bibliotheca Diatomologica. Vol. 29. P. 84–85. Pl. 52. Figs 29–36.

**Comments:** The specimens in our sample were 10.4–13.6 μm long, 4.2–5.1 μm wide, and had 20 striae in 10 μm and 35–40 areolae in 10 μm.

**Figure 10:**
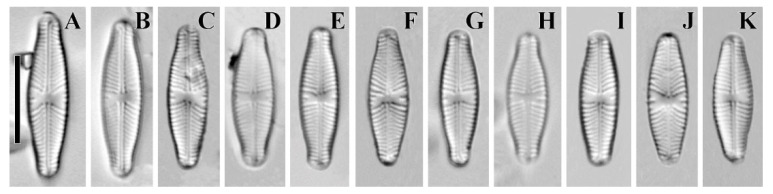
(**A**–**K**) *Cymbosellaphora geisslerae* (Jahn) Kulikovskiy, Glushchenko, Genkal, and Kociolek comb. nov. Light microscopy, differential interference contrast, size diminution series. Mongolia. Slide no. 02642 (**A**–**C**,**E**–**G**,**I**–**K**); 02684 (**D**,**H**). Scale bar = 10 μm.

**Figure 11:**
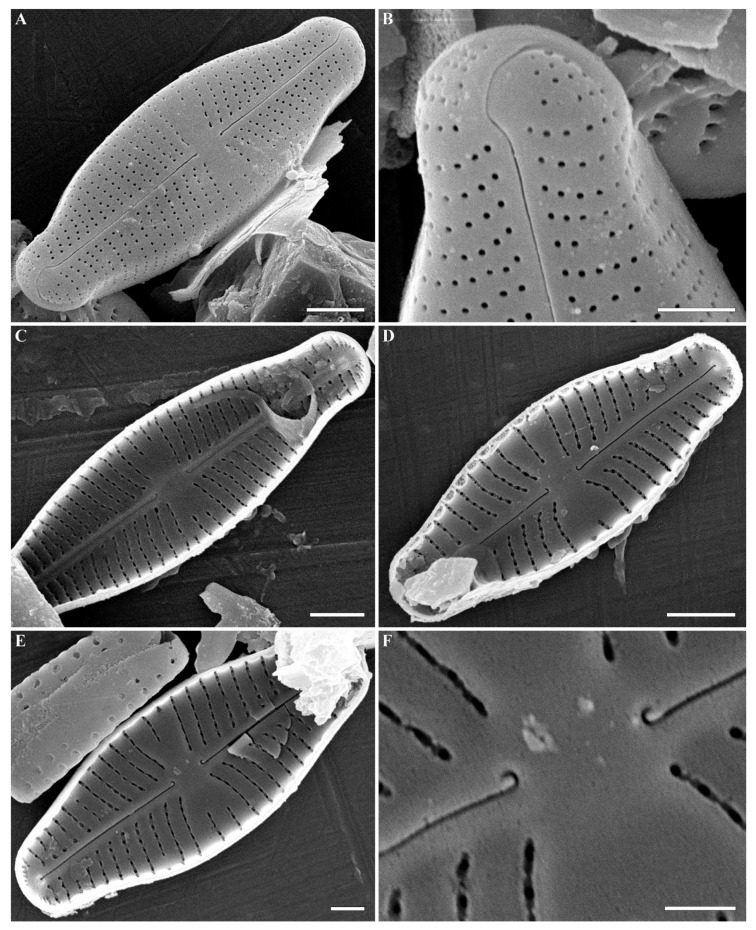
(**A**–**F**) *Cymbosellaphora geisslerae* Kulikovskiy, Glushchenko, Genkal and Kociolek comb. nov. Scanning electron microscopy. (**A**,**B**) External views. (**C**–**F**) Internal views. (**A**–**E**) The whole valve. (**B**) Valve end. (**F**) Central area. Tectula, as small struts, are present between areolar openings within each stria. Scale bar (**A**,**C**,**D**) = 2 μm; (**B**,**E**) = 1 μm; (**F**) = 0.5 μm.

*Cymbosellaphora geisslerae* (Jahn) Kulikovskiy, Glushchenko, Genkal and Kociolek comb. nov. (**Figure 10 and Figure 11**)

**Basionym:** *Navicula geisslerae* Jahn 1992. Diatom Research. Vol. 7. Issue 1. P. 69–75. Figs 1–15.

**Synonym:** *Naviculadicta geisslerae* (Jahn) Jahn in Lange-Bertalot and Moser 1994.

**Comments:** The specimens in our sample were 14.3–18.8 μm long, 4.3–4.8 μm wide, and had 19–21 striae in 10 μm and 38–40 areolae in 10 μm.

*Cymbosellaphora laterostrata* (Hustedt) Kulikovskiy, Glushchenko, Genkal and Kociolek comb. nov.

**Basionym:** *Navicula laterostrata* Hustedt 1925. Bacillariales aus Schlesien. II. Nachtrag. Internationale Revue der gesamten Hydrobiologie und Hydrographie. Band 13. S. 345–357.

**Synonym:** *Navicula mournei* R.M. Patrick 1959.

All of the species discussed herein, transferred to the genus *Cymbosellaphora* gen. nov., i.e., *C. vietnamensis* sp. nov., *Cymbosellaphora absoluta* comb. nov., *Cymbosellaphora circumborealis* comb. nov., *Cymbosellaphora geisslerae* comb. nov., *Cymbosellaphora laterostrata* comb. nov., share the same morphological features. The feature that they all share, and which distinguishes them from *Sellaphora*, is the internal pore occlusions. In these taxa, areolae are covered internally by a nonperforated silica layer with presence of rectangular struts that divide every areola. During the cleaning of the valves, these rectangular struts can be destroyed, revealing two outgrowths situated parallel to the valve level and opposite one another. The presence of nonperforated silica covers, as well as struts and outgrowths, are similar to the pore occlusions of *Placoneis* described by Cox [23]. Differences between *Cymbosellaphora* gen. nov. and *Placoneis* include the number of vertical points around the areola internally that are covered by a flap-like covering (tectulum, “little silica roof”) in the sense of Cox [23]. However, these two genera are similar, based on the presence of two outgrowths situated parallel to valve level and opposite one another (see Kulikovskiy et al.) [27]. This morphological feature is present in every species assigned to our new genus, suggesting that *Cymbosellaphora* gen. nov. is characterized by pore occlusions that are like the tectulum of cymbelloid diatoms, not the hymenate occlusions characteristic of *Navicula* or *Sellaphora*. On this basis, we assign our new genus to the family Cymbellaceae Greville. This family, according to Kulikovskiy et al. [12], includes genera with almost naviculoid symmetry, such as *Cymbellafalsa* Lange-Bertalot and Metzeltin; *Geissleria* Lange-Bertalot and Metzeltin; *Khursevichia* Kulikovskiy, Lange-Bertalot and Metzeltin; *Ochigma* Kulikovskiy, Lange-Bertalot and Metzeltin; *Paraplaconeis* Kulikovskiy, Lange-Bertalot and Metzeltin; *Placoneis* and *Rexlowea* Kociolek and Thomas.

The new genus, *Cymbosellaphora* gen. nov., shares morphological features such as the tectulum, naviculoid symmetry with slight dorsiventrality, and perforated girdle bands with cymbelloid diatoms [25,26]. However, genera in the cymbelloid lineage are distinguished from each other by slightly different pore structures.

All taxa have pore occlusions covered internally by flap-like coverings called tectula, but the presence or absence of different struts, the shape of the tectulum, and the number and aperture morphology are different. For example, *Geissleria* is a very similar genus to *Cymbosellaphora* gen. nov., but it differs from it by the presence of two broad outgrowths situated parallel to valve level and opposite one another ([12], Figure 2.14.6; 41). Additionally, *Geissleria* is characterized by the presence of special areolae at each end, called an annulus [28]. *Paraplaconeis* is characterized by having a much more complicated areolar structure than *Cymbosellaphora* gen. nov. and *Placoneis*. *Paraplaconeis* has uniform slits that have a stalked flap and long struts separating the areolae internally, forming a polygonal pattern over the alternating biseriate areolae of the valve face [27]. Areolae are also covered by tectula internally (see discussion in Kulikovskiy et al.) ([12], Figure 2.6.2). *Rexlowea* is characterized also by having naviculoid symmetry, but this genus possesses a prominent undulate raphe and striae with internal occlusions with narrow bases and flared tips protruding into the areolae [12,29,30]. *Khursevichia* differs by having areolae with noticeable oval flat hollows—circuses, on which the small outgrowths are present [31]. These outgrowths link up with their tops, closing the opening of the areolae. Pore occlusions of *Ochigma* are much more complicated and characterized by large areolae with circular hollows. Each areola has a flat circus, going deep into the valve with a tapered round or oblong hole in the form of a funnel. This circus-like plane has a large number of rather long, irregularly shaped nodules, which have a silica layer on the top [31].

*Cymbellafalsa* is a genus very similar to *Cymbosellaphora* gen. nov. *Cymbellafalsa* was described by Lange-Bertalot and Metzeltin in 2009 from Mongolian material [32]. Species assigned to this genus were previously members of the genera *Cymbella* and *Navicula*. The referral to *Cymbella* was based on the visible dorsiventral shape and the presence of pore fields at both valve ends. The presence of pore fields is an important morphological feature that divides *Cymbellafalsa* from *Cymbosellaphora* gen. nov. Pore fields in *Cymbellafalsa* are characterized by the presence of an undulate flap–like silica strip above the internal apertures of each pervalvar row of foramina, not completely occluding internal apertures ([32], Figure 266: 4). Members of *Cymbosellaphora* gen. nov. have slightly compressed striae near the valve ends but never with the special flap-like silica strips seen in *Cymbellafalsa*. The latter genus is similar to *Cymbosellaphora* gen. nov., based on striae structure and pore occlusion type. However, valve shape and striae structure are not helpful features for combining genera relative to morphological variation associated with pore occlusions. This situation was discussed and is shown with respect to the genus *Karthickia* [33,34]. Our new genus is easily distinguished from *Navicula* and *Sellaphora* by the internal absence of hymenes.

The taxonomic position of species now assigned to the genus *Cymbosellaphora* gen. nov. was discussed previously. Jahn [25], who described *C. geisslerae* comb. nov. as a member of *Navicula*, discussed the similarity of this species with the genus *Placoneis*. Jahn [25] undertook a comprehensive morphological investigation of this small-celled, widespread species and showed it to have pore occlusions that are typical for Cymbellaceae. However, the description of the tectulum and its differences from other pore occlusions of naviculoid genera was not given until later [23]. Our investigations of different cymbelloid genera, using molecular data as well as morphological data, showed that pore occlusions are an important morphological feature for differentiating between cymbelloid and naviculoid genera [20,35]. The description of new genera with scanning electron microscopy reveals that pore occlusions are different between cymbelloid genera. The structure of the tectulum across cymbelloid genera differs mainly by presence/absence, as well as the number and shape of struts into the areolae. This morphological feature will receive further investigation.

*C. vietnamensis* sp. nov., *C. absoluta* comb. nov., *C. circumborealis* comb. nov., *C. geisslerae* comb. nov. and *C. laterostrata* comb. nov. can easily be distinguished from each other. The morphologies of these species are summarized in Table 1.

Morphological investigation of the populations of the species studied herein are similar to previous descriptions [25,36,39,40,41,42]. Chudaev and Gololobova [41] prepared a comprehensive light and scanning electron investigation of *C. geisslerae* comb. nov. ([41]: Figs 229: 1–35 and 230: 1–5) and *C. laterostrata* comb. nov. ([41]: Figs 231: 1–6 and 232: 1–14]). The populations of the species studied herein share morphological features documented in the literature cited above.

The species *Cymbosellaphora vietnamensis* sp. nov. is known only from the type locality (Lake Ba Bể, Vietnam). This is the first documented finding of this genus in Southeast Asia. Reports of *Navicula absoluta* and *Navicula laterostrata* in Indonesia are not supported by illustrations [43,44].

All other documented species of the genus are known from Holarctic habitats. *Cymbosellaphora absoluta* comb. nov. was described as *Navicula absoluta* from Northern Germany (from Lake Garrensee) [36]. The species was also previously found in Mongolia, in the sediments of the Great Lakes Depression, sine loco [38]. We recorded this species in the benthos, periphyton, and sediments of Lake Davaa, as well as in the periphyton of the unnamed river flowing out of Lake Davaa.

*Cymbosellaphora circumborealis* comb. nov. was described as *Navicula circumborealis* from Lake James, Canada [40]. The species is known from Sweden, Finland, and the Aleutian Islands [40]. In Mongolia, this species was found by us in the periphyton and benthos of Boreug Bay and Heeguer Bay of Lake Khövsgöl.

*Cymbosellaphora geisslerae* comb. nov. was described as *Navicula geisslerae* from Spree River, Berlin [39]. This species was also illustrated from Lake James, Canada, Central Europe [39,40], and from Lake Glubokoe, Russia [41]. This species was recorded by us from Mongolia, from the benthos of Khövsgöl Lake (Heeguer Bay), as well as from Unnamed Lake near Khövsgöl Lake, separated by a sandbar.

*Cymbosellaphora laterostrata* comb. nov. was invalidly described as *Navicula laterostrata* from Germany and reported from lakes Schlawasee, Ogglisch-Mühlensee, Kleiner Tarnauersee [42]. Later, Reimer Simonsen validated this taxon [37]. It has also been illustrated from Großes Heiliges Meer lake [40]. The species was found in Russia, in Lake Glubokoe [41]. This species was also previously found in Mongolia, in the sediments of Har Us Lake, Dood Lake, Davaa Lake, and Terhiyn Tsagaan Lake [38]. This species was not noted in our material for the present report.

## 3. Conclusions

Review of taxa previously transferred from *Navicula*, *Naviculadicta*, and *Eolimna* to *Sellaphora* show they have features that are found in the genera and species of the Cymbellales rather than the Naviculales. The tectula morphology in these taxa are different from other genera in Cymbellales, warranting the creation of a new genus for them; thus, the genus *Cymbosellaphora* is proposed to accommodate these species with unique tectular morphology. The genus is found in the Holarctic but also from Vietnam. A further review of the structure of tectula is warranted in order to document further variability in its structure and its potential to diagnose natural groups. Further research on small species recently allocated to the genus *Sellaphora* may uncover additional cryptic diversity in this catch-all genus.

## 4. Materials and Methods

**Sampling**. A list of all samples examined in this study, along with their geographic position, is presented in Table 2.

**Preparation of slides and microscope investigation**. Samples were processed by means of a standard procedure involving treatment with 10% HCl and concentrated hydrogen peroxide. After treatment with HCl, the samples were washed with deionized water. Permanent diatom preparations were mounted in Naphrax^®^ (Brunel Microscopes, Chippenham, UK). Light microscopic (LM) observations were performed with a Zeiss Axio Scope A1 (Zeiss, Oberkochen, Germany) microscope equipped with an oil immersion objective (×100/n.a.1.4, DIC). For scanning electron microscopy (SEM), parts of the suspensions were fixed on aluminum stubs after air drying. The stubs were sputter-coated with 50 nm of gold in an Eiko IB 3 (Eiko Engineering, Yamazaki, Hitachinaka Shi, Ibaraki Ken, Japan). Valve ultrastructure was examined by means of a JSM-6510LV scanning electron microscope (JEOL Ltd., Akishima, Tokyo, Japan), operated at 10 kV and 11 mm distance.

Samples and slides are deposited in the collection of Maxim Kulikovskiy at the Herbarium of the Institute of Plant Physiology Russian Academy of Sciences, Moscow, Russia.

## Figures and Tables

**Table 1:** Comparison of *Cymbosellaphora vietnamensis* sp. nov. and four species transferred to *Cymbosellaphora* gen. nov.

	*Cymbosellaphora vietnamensis* sp. nov.	*Cymbosellaphora absoluta* comb. nov.	*Cymbosellaphora circumborealis* comb. nov.	*Cymbosellaphora geisslerae* comb. nov.	*Cymbosellaphora laterostrata* comb. nov.
**References**	This study	[36,37,38,39]; This study	[40]; This study	[25,40,41]; This study	[37,38,40,41,42]
**Distribution**	Vietnam; Ba Bể Lake	Holarctic; Central Europe; Mongolia	Holarctic: Europe; North America; Canada; Mongolia	Europe; Canada; Russia	Europe; Russia; Mongolia
**Outline**	Almost liner to linear–elliptic, naviculoid, slightly dorsiventral in longer cells with broadly protracted ends	Elliptic–lanceolate with drawn out, obtusely rounded rostrate to subcapitate ends	Linear–lanceolate or elliptical–lanceolate or lanceolate	Narrow–elliptical with protracted and broadly rounded poles	Linear–elliptic with subcapitate ends
**Axial area**	Narrow, straight	Narrow	Very narrow	Narrow	Narrow
**Central area**	Variable, small to medium-size, transversely widened and irregularly delimited	Variable, small-to-medium size, transversely widened, and irregularly delimited	Transversely expanded and bordered by 1, 2, and rarely 3 very short striae	Transversely expanded and bordered by 1–4 very short striae	Variable, formed by short striae
**Valve length (μm)**	10.7–31.4	10–20	9–18	8.9–27.6	22.9–31.0
**Valve breadth (μm)**	5.0–6.7	4.5–6.0	4.2–5.5	4.0–6.6	7.1–8.8
**Striae in 10 μm**	14–16	25–28	17–20	18–25	18.5–20.4
**Areolae in 10 μm**	35–40	25–30	35–40	30.7–38.7	24.8–31.1

**Table 2:** List of the collected samples and their characteristics.

Sample No	Slide No	Locality	Coordinates	Substratum	Cond., µS/cm	pH	t, °C	Collection of Date
**Vietnam**								
BB9	02151	Ba Bể Lake, Bắc Kạn Province	22°23.605′ N; 105°36.856′ E	benthos a depth of 3 m	26	8.5	174	29 April 2015
**Mongolia**								
Mn079	02431	Davaa Lake	48°10.803′ N; 98°46.107′ E	benthos	20	8.1	13	8 July 2015
Mn088	02440	Davaa Lake	48°10.827′ N; 98°45.828′ E	sand	20	6.9	13	8 July 2015
Mn089	02441	Davaa Lake	48°10.827′ N; 98°45.828′ E	sand	20	6.9	13	8 July 2015
Mn090	02442	Davaa Lake	48°10.827′ N; 98°45.828′ E	stone and sand	20	6.9	13	8 July 2015
Mn093	02445	Davaa Lake	48°10.827′ N; 98°45.828′ E	periphyton	20	6.9	13	8 July 2015
Mn094	02446	Davaa Lake	48°11.145′ N; 98°45.746′ E	bottom sediment a depth of 2.5 m	38	7.7	15	8 July 2015
Mn095.1	02447	Davaa Lake	48°11.145′ N; 98°45.746′ E	bottom sediment a depth of 4 m	38	7.7	15	8 July 2015
Mn095.5	02451	Davaa Lake	48°11.145′ N; 98°45.746′ E	bottom sediment a depth of 4 m	38	7.7	15	8 July 2015
Mn104	02457	Unnamed river flowing out of the Davaa Lake	48°10.791′ N; 98°46.272′ E	periphyton	38	7.7	15	8 July 2015
Mn289.1	02642	Unnamed Lake near the Khövsgöl Lake, separated by a sandbar	50°59.165′ N; 100°42.514′ E	bottom sediment a depth of 5 m	100	8.9	18	28 July 2015
Mn312	02663	Khövsgöl Lake	50°47.215′ N; 100°31.837′ E	periphyton	243	8.6	12	28 July 2015
Mn335.8	02684	Khövsgöl Lake, Heeguer Bay	50°38.641′ N; 100°31.397′ E	benthos	304	10.6	22	22 July 2015
Mn308	02695	Khövsgöl Lake	50°47.215′ N; 100°31.837′ E	periphyton	243	8.6	12	28 July 2015
Mn283.2	03009	Khövsgöl Lake, Boreug Bay	50°59.380′ N; 100°42.507′ E	benthos	236	8.7	11.5	21 July 2015

## Data Availability

Data are contained within the article.
